# Pathologic Complete Response Following Frailty-Adjusted Neoadjuvant Paclitaxel in an 88-Year-Old Patient With Radiation-Associated Breast Angiosarcoma

**DOI:** 10.7759/cureus.113741

**Published:** 2026-07-31

**Authors:** Alyssa C Brown, Ajay McLaughlin, Kaylyn M Lazuka, Devapiran Jaishankar, Natalie R Scott

**Affiliations:** 1 Department of Surgery, East Tennessee State University, Johnson City, USA; 2 Department of Medical Oncology, The US Oncology Network, Johnson City, USA

**Keywords:** angiosarcoma, breast angiosarcoma, mastectomy, neoadjuvant chemotherapy, paclitaxel, pathologic complete response, radiation-associated angiosarcoma, soft tissue sarcoma

## Abstract

Radiation-associated breast angiosarcoma is a rare but highly aggressive malignancy with limited evidence to guide optimal management in elderly patients. We present the case of an 88-year-old woman with a history of stage IIIA estrogen receptor-positive invasive ductal carcinoma of the right breast treated with breast-conserving surgery, chemotherapy, radiation therapy, and endocrine therapy in 2014. Approximately 11 years after completing radiation therapy, she developed progressive right breast firmness, violaceous skin discoloration, and persistent ecchymotic changes concerning for vascular malignancy. Imaging demonstrated diffuse skin thickening without a discrete mass, and punch biopsy of skin changes confirmed high-grade angiosarcoma with MYC amplification. Staging studies showed no distant metastatic disease. Given her advanced age, frailty, and comorbidities, she underwent neoadjuvant weekly paclitaxel with initial dose reduction followed by response- and tolerance-guided dose escalation. Despite treatment interruptions related to frailty and fall-related injuries, she completed 12 cycles and subsequently underwent right simple mastectomy. Final pathology demonstrated no residual angiosarcoma, consistent with a pathologic complete response. This case highlights the potential efficacy of frailty-adjusted neoadjuvant paclitaxel in elderly patients with radiation-associated breast angiosarcoma and supports individualized neoadjuvant strategies for patients who are not candidates for standard multi-agent chemotherapy.

## Introduction

Radiation-associated breast angiosarcoma (RABA) is a rare but increasingly recognized complication of breast-conserving therapy, typically presenting 5-10 years after radiation exposure [1]. It is characterized by aggressive behavior and poor prognosis, with limited prospective data guiding optimal treatment strategies [1]. The cumulative incidence of thoracic soft tissue sarcoma following breast radiation therapy has been estimated at 0.15-0.2% at 10 years, with radiation exposure representing the strongest known risk factor for RABA development (RR 5.5-8.1 compared with non-irradiated patients) [2,3]. RABA typically presents as violaceous, ecchymotic, or bruise-like patches, plaques, or nodules within previously irradiated skin. Because these findings often resemble benign post-traumatic or radiation-induced skin changes, diagnosis is often delayed [3]. Paclitaxel has demonstrated activity against angiosarcoma and is considered a preferred systemic agent in metastatic disease according to the National Comprehensive Cancer Network (NCCN) Soft Tissue Sarcoma Guidelines [4]. However, specific data regarding neoadjuvant dose-modified regimens, particularly in elderly or frail patients, remain sparse, in part because of the rarity of the disease [2-4]. We present the case of an 88-year-old woman with RABA successfully treated with frailty-adjusted neoadjuvant paclitaxel who achieved a pathologic complete response (pCR), highlighting the potential efficacy of individualized taxane-based therapy in this vulnerable patient population.

## Case presentation

An 88-year-old woman with multiple comorbidities, classified as mildly frail (Clinical Frailty Scale 5 [5]), presented with progressive firmness and discoloration of the right breast following a ground-level fall. She initially attributed these symptoms to trauma; however, the persistence and progression of ecchymosis over several months as well as generalized shrinking and hardening of her right breast raised concern for an underlying malignancy. Her past medical history was significant for stage IIIA (pT1, pN2a) invasive ductal carcinoma of the right breast diagnosed in September 2014, at which time she underwent breast-conserving surgery with axillary lymph node dissection (8 of 11 nodes positive). This was followed by dose-dense doxorubicin and cyclophosphamide, adjuvant paclitaxel, and completion of radiation therapy in July 2015, intensity-modulated radiation therapy 45Gy with boost to 63Gy, 45Gy to lymph node basins. She subsequently received 10 years of adjuvant endocrine therapy with anastrozole.

**Figure 1:**
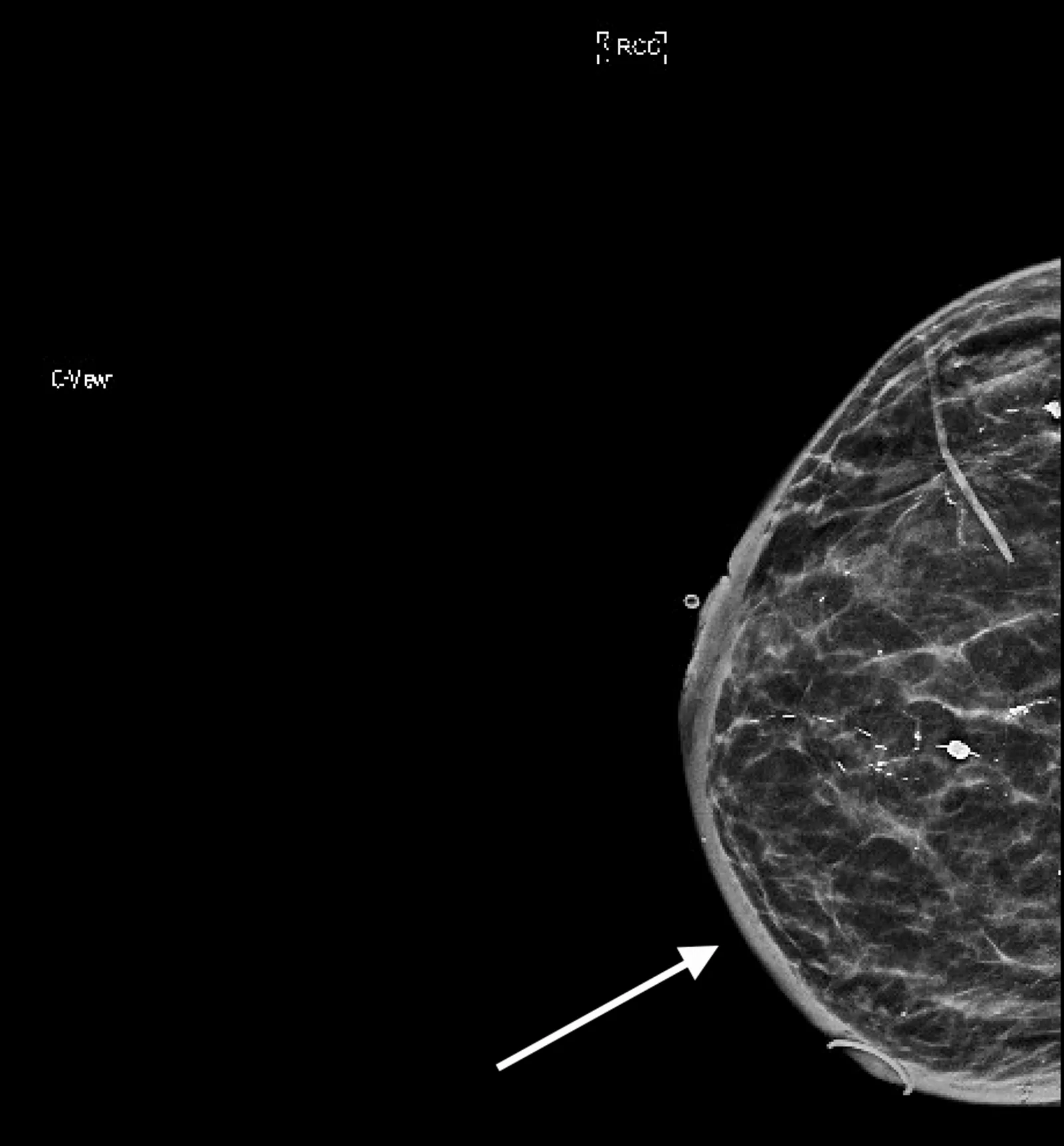
Pretreatment mammogram Craniocaudal mammographic view of the right breast demonstrating diffuse skin thickening without a discrete dominant mass, consistent with the patient's extensive cutaneous involvement.

**Figure 2:**
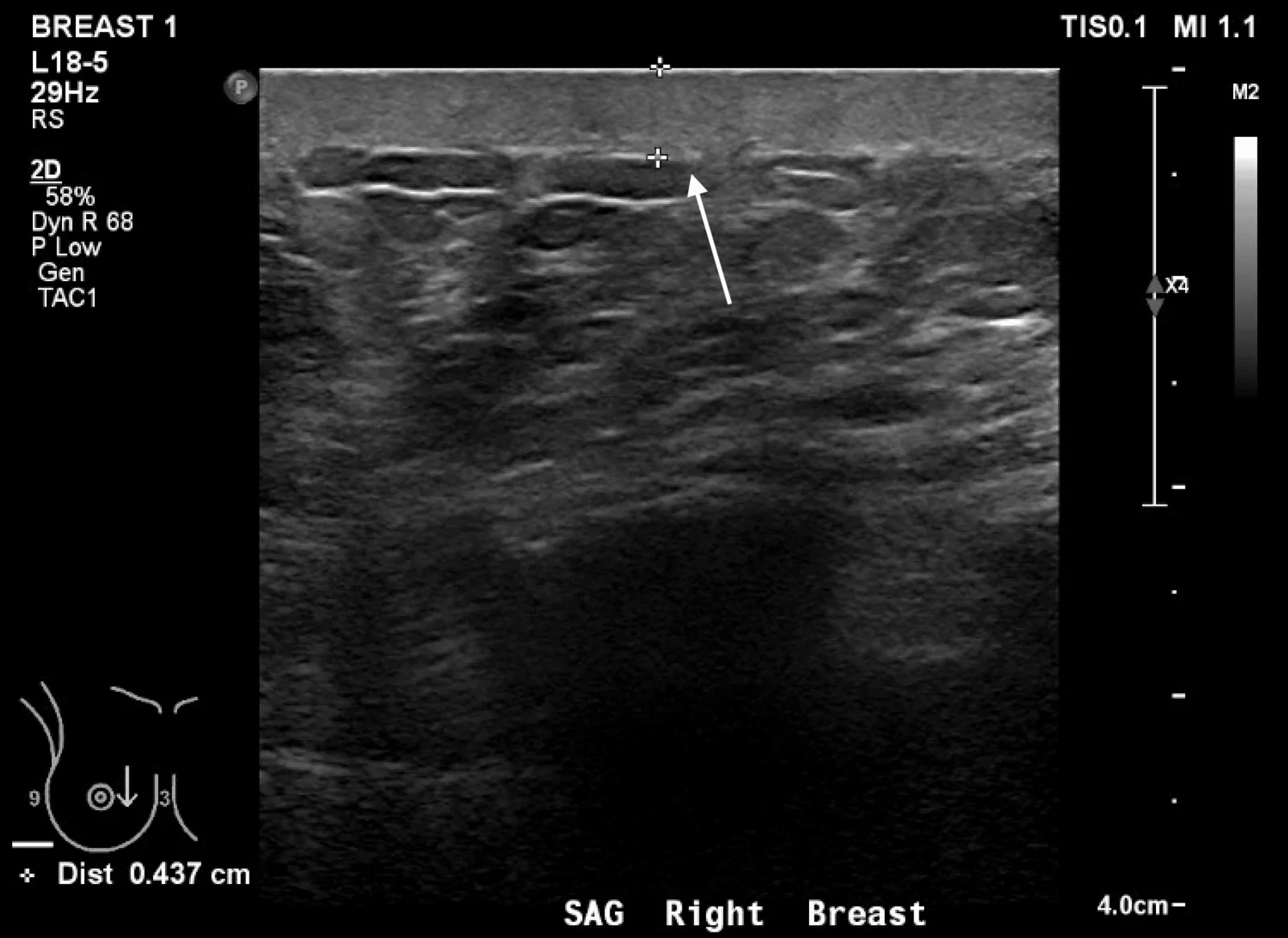
Pretreatment ultrasound Targeted ultrasound of the right breast demonstrating abnormal skin thickening, corroborating the clinical findings of diffuse skin involvement.

Approximately 11 years after breast-conserving therapy, she noted progressive breast heaviness, firmness, and persistent ecchymosis, which was initially attributed to a recent ground-level fall. Subsequent diagnostic mammography (Figure 1) and ultrasound (Figure 2) demonstrated diffuse skin and trabecular thickening without a discrete underlying mass (BI-RADS 4). Physical examination demonstrated extensive violaceous discoloration of the right breast extending from the infraclavicular region to the inframammary fold, with skin changes encompassing most of her residual breast tissue, most prominently medially. Skin involvement was sufficiently extensive that upfront mastectomy was not considered surgically feasible. In addition, her right breast was noted to be significantly smaller than her left breast and very firm. There was no axillary adenopathy.

A right breast skin punch biopsy performed in September 2025 revealed high-grade angiosarcoma. Immunohistochemistry was positive for CD31, CD34, and D2-40, and negative for ER, PR, HER2, GATA3, and cytokeratin markers. Ki-67 demonstrated a markedly elevated proliferation index, and fluorescence in situ hybridization confirmed MYC (8q24) amplification, consistent with RABA. Staging positron emission tomography/computed tomography demonstrated hypermetabolic activity within the right breast and skin thickening without evidence of distant metastatic disease.

The patient’s case was reviewed at a multidisciplinary tumor board. As she was not a surgical candidate because negative margins and primary closure were not considered achievable with upfront surgery, the recommendation was to proceed with neoadjuvant weekly paclitaxel. Given her advanced age and frailty, therapy was initiated at 75% of the standard weekly dose (approximately 60 mg/m², based on the standard weekly dose of 80 mg/m²) [4] for the first three cycles, with good clinical and hematologic tolerance. Given her early clinical response, dosing was escalated to approximately 80% of the standard dose for cycles 4-6 (approximately 64 mg/m²), followed by escalation to 90% of the standard dose beginning with cycle 7 (approximately 72 mg/m²). Treatment was complicated by another traumatic fall resulting in a nondisplaced scapular fracture requiring hospitalization and rehabilitation. Despite this, the patient elected to continue neoadjuvant therapy. She subsequently completed cycles 8-10 at the full standard dose (80 mg/m²), followed by dose reduction during cycles 11-12 because of her overall clinical status. Throughout treatment, she was evaluated monthly by surgical oncology and demonstrated marked clinical and radiographic response, including softening of the breast to near-normal consistency with near-complete resolution of the overlying cutaneous discoloration.

Final pathology demonstrated no definite residual angiosarcoma, consistent with a pathologic complete response. The mastectomy specimen, including the skin and breast tissue, was extensively sampled (48 tissue sections) and demonstrated treatment-related dermal fibrosis, fibrous change, and focal fat necrosis without definitive viable tumor. Surgical margins were negative. A single microscopic focus (<0.5 mm) of atypical cells could not be definitively characterized despite immunohistochemical evaluation. This was favored to represent treatment-related reactive atypia, although a minute focus of residual angiosarcoma could not be completely excluded. At three months postoperatively, she remained without clinical or radiographic evidence of local or distant recurrence and continues routine surveillance with her breast surgical oncology team.

## Discussion

RABA is an uncommon late complication of breast-conserving therapy, most commonly developing 5-10 years after radiation exposure [1,4]. In a systematic review of 222 patients, Depla et al. reported a median latency of 5-10 years [1], while Veiga et al., in a retrospective cohort of more than 450,000 breast cancer survivors, identified prior radiation therapy as the strongest risk factor for subsequent thoracic soft tissue sarcoma (RR 5.5) [2]. The 11-year interval observed in our patient is therefore consistent with the expected natural history of RABA and reinforces the importance of maintaining a high index of suspicion in patients presenting with persistent violaceous skin changes, ecchymosis, or breast firmness following prior breast-conserving therapy.

Management of RABA remains challenging due to its rarity and the lack of prospective randomized data to guide treatment [1,3,6,7]. Consequently, no standardized treatment protocol has been established, and treatment recommendations are largely derived from retrospective series and institutional experience [3,6,7]. Radical surgical resection remains the cornerstone of therapy, although postoperative hemorrhagic complications are well-recognized in these highly vascular tumors [4,6]. More recently, retrospective studies have demonstrated that neoadjuvant taxane-based chemotherapy, alone or as part of multimodality therapy, has been associated with pathologic complete response rates of 69-75% and improved local control, though prospective validation is still needed to define optimal patient selection and treatment sequencing [6-8]. Our patient's clinical course extends this emerging body of evidence, as neoadjuvant paclitaxel successfully converted initially unresectable disease into a pCR following surgical resection.

This case highlights several important clinical considerations. At presentation, the extent of cutaneous and breast involvement precluded margin-negative resection with primary wound closure, making neoadjuvant therapy essential to achieve surgical resectability [6,8]. The patient's advanced age and frailty further complicated management, limiting the feasibility of standard multi-agent chemotherapy and necessitating an individualized treatment strategy [7]. Despite initiating weekly paclitaxel at a substantially reduced dose, she demonstrated an early clinical response that permitted progressive dose escalation while maintaining tolerability. Ultimately, she achieved a pCR following neoadjuvant paclitaxel, consistent with the favorable response rates reported in recent neoadjuvant taxane series [8]. However, unlike previously reported cohorts, our patient achieved this outcome despite advanced age, significant frailty, and an initially dose-reduced regimen, suggesting that carefully titrated, response-adapted neoadjuvant therapy may be an effective strategy for select elderly patients who are not candidates for conventional treatment.

At the most recent follow-up (three months after surgery), the patient remained without clinical or radiographic evidence of local or distant recurrence. However, the available follow-up remains relatively short, and continued long-term surveillance is warranted given the high risk of recurrence associated with RABA. These findings suggest that frailty-adjusted paclitaxel can retain meaningful therapeutic efficacy in RABA, particularly in patients who are not candidates for more aggressive regimens. These findings have important implications for individualized treatment strategies in elderly populations, where balancing efficacy and tolerability is critical. This case also highlights a pragmatic approach to dose-modified chemotherapy in frail elderly patients. Rather than employing a fixed dose reduction, we used a response-adapted strategy with gradual dose escalation based on clinical tolerance and early treatment response. This approach enabled delivery of near full-intensity therapy while minimizing toxicity. Such a strategy may be particularly valuable in elderly patients with limited physiologic reserve, for whom standard dosing may be poorly tolerated yet excessive dose attenuation risks compromising oncologic efficacy. In this case, response-adapted dosing facilitated conversion to surgically resectable disease while preserving the patient's quality of life throughout treatment.

## Conclusions

This case suggests that frailty-adjusted, response-adapted neoadjuvant paclitaxel may achieve meaningful oncologic response, including pCR, in selected frail elderly patients with RABA. While the favorable response observed in this single case may not be generalizable, a stepwise dose-escalation strategy based on clinical tolerance may allow safe delivery of effective therapy in carefully selected patients who are not candidates for standard multi-agent regimens. As breast-conserving surgery with radiation therapy continues to expand and breast cancer survivorship improves, the incidence of delayed radiation-associated malignancies, such as RABA, may increase. Further study with larger cohorts and longer follow-up is needed to define optimal patient selection and treatment sequencing for frailty-adapted neoadjuvant strategies.
